# Advancements and challenges in immunotherapy for gastric cancer: current approaches and future directions

**DOI:** 10.3389/fimmu.2025.1592733

**Published:** 2025-05-21

**Authors:** Wenyu Zhang, Jingzheng Chen, Zehao Wei, Jiaqian Song, Xinyi Zha, Deqiang Wang, Min Xu

**Affiliations:** ^1^ Department of Gastroenterology, Affiliated Hospital of Jiangsu University, Zhenjiang, China; ^2^ Department of Cardiology, Affiliated Hospital of Jiangsu University, Zhenjiang, China; ^3^ Department of Rheumatology, Affiliated Hospital of Jiangsu University, Zhenjiang, China; ^4^ Department of Oncology, Digestive Disease Institute & Cancer Institute of Jiangsu University, Affiliated Hospital of Jiangsu University, Zhenjiang, China; ^5^ Institute of Digestive Diseases, Jiangsu University, Zhenjiang, China

**Keywords:** gastric cancer, immunotherapy, tumor microenvironment, immune checkpoint inhibitors, adoptive cell therapy, tumor vaccines, oncolytic viruses

## Abstract

Gastric cancer (GC) poses a major global health challenge, marked by high incidence and mortality rates. Conventional treatments such as surgery, chemotherapy, and targeted therapies show limited effectiveness in patients at advanced stages. As a result, immunotherapy has emerged as a promising strategy in the battle against cancer. In recent years, immunotherapy has flourished, with immune checkpoint inhibitors becoming widely applied in GC, while other immunotherapies are also rapidly advancing in clinical development, providing new therapeutic options for patients. The introduction of immunotherapy has profoundly changed the approach to GC treatment, with the expectation that additional immunotherapies will be developed in the near future. However, the clinical effectiveness of these therapies remains constrained due to the complexity of the tumor microenvironment (TME) in GC, the significant heterogeneity among patients, as well as the occurrence of immune therapy resistance and adverse reactions. This review provides an overview of recent advancements in GC immunotherapy, focusing on ICIs, adoptive cell therapy, and tumor vaccines. Key challenges such as patient selection, biomarker development, and combination therapy optimization are also discussed. In the future, a deeper exploration of the TME characteristics of GC and the implementation of personalized and precise immunotherapy are expected to further improve therapeutic outcomes and patient prognosis.

## 1 Introduction

**Figure 1:**
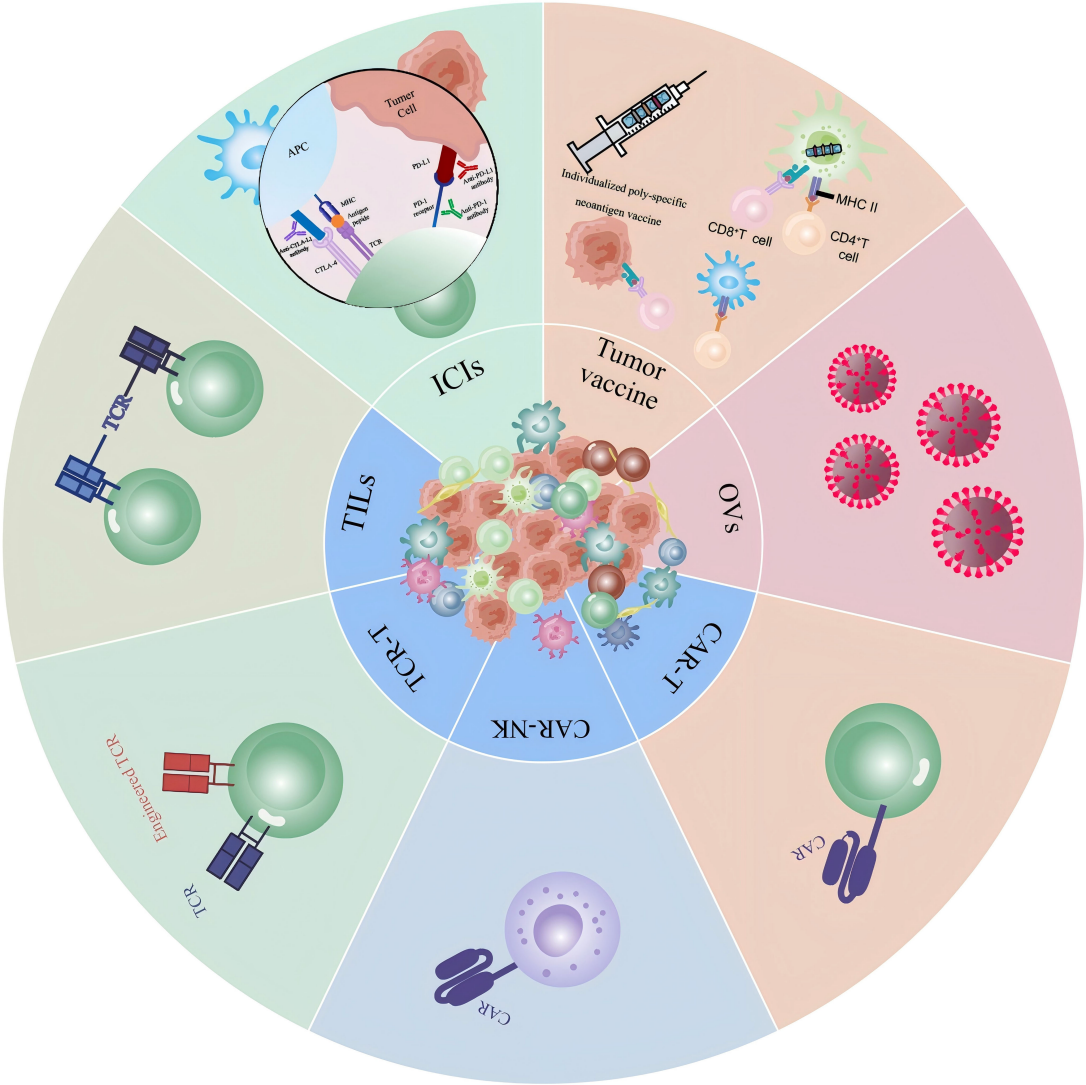
Novel immunotherapy techniques in gastric cancer: A visual summary of various immunotherapeutic strategies for gastric cancer and their mechanisms of action in cancer treatment. Includes ICIs, various therapeutic modalities for ACT, tumor vaccine therapies, and lysovirus therapies.

Cancer is a major global health challenge, representing a significant risk to human health and survival. The incidence of gastrointestinal cancers, which exhibit diverse epidemiological backgrounds along with genetic and epigenetic aberrations, continues to rise, representing a global trend. This has led to gastrointestinal cancers becoming among the most prevalent cancers worldwide, accompanied by a typically high mortality rate (1). Gastric cancer (GC) is a widespread malignancy within the digestive system, with its incidence and mortality rates are the fifth highest in the global rankings (2). The occurrence of GC is associated with various risk factors, including Helicobacter pylori infection, atrophic gastritis, smoking, high salt intake, and hereditary diseases (3–5). For early - stage GC, surgical resection is still the principal treatment option. However, the lack of clear clinical signs causes most GC patients to be diagnosed in later stages. Although chemotherapy has improved the survival rate and quality of life for individuals with locally advanced, unresectable, or metastatic GC, the prognosis remains unsatisfactory (6, 7). GC exhibits complex molecular subtypes and significant heterogeneity, leading to overall suboptimal treatment efficacy (8, 9). In recent years, with the advancements in molecular biology and tumor biology, the application of immunotherapy in the treatment of GC has been expanding (10). Tumor immunotherapy, including immune checkpoint inhibitors (ICIs), Adoptive Cell Therapy (ACT), Cancer Vaccines and other therapies, has become a new approach to cancer treatment (**Figure 1**). The main emphasis of tumor immunotherapy is immune cells, with the goal of eradicating tumor cells by boosting the body’s inherent immune response, reshaping the immune microenvironment (IME), and utilizing other mechanisms, all while minimizing damage to healthy cells (11, 12). The progression of GC is closely related to the tumor microenvironment (TME) (13). Grasping the evolution of the GC microenvironment and the mechanisms by which it adapts to therapeutic pressure is vital for formulating effective treatment plans (14). In addition, HER2 and other targets have proven to play a crucial role in the management of GC. The large international multicenter HER-EAGLE study indicates that the global HER2-positive rate in GC is approximately 10% to 20% (15). In recent years, breakthrough advancements have been made in the combination of immunotherapy and targeted therapy in GC treatment, providing more therapeutic options for GC patients.

ICIs restore the antitumor activity of T cells, thereby enhancing the immune system’s ability to recognize and target tumors. Although ICIs have been shown to be effective in various cancer types, challenges remain when applied to GC. These challenges include the tumor’s heterogeneity, the complexity of immune evasion mechanisms, and the diversity of immune cells within the TME. This article will discuss the components and functions of the GC microenvironment, review the recent advancements in immunotherapy for GC, and analyze the key issues encountered in current clinical practice. Furthermore, it will provide perspectives on future directions, offering valuable references for researchers and clinicians in the field of GC immunotherapy.

## 2 TME of GC

**Figure 2:**
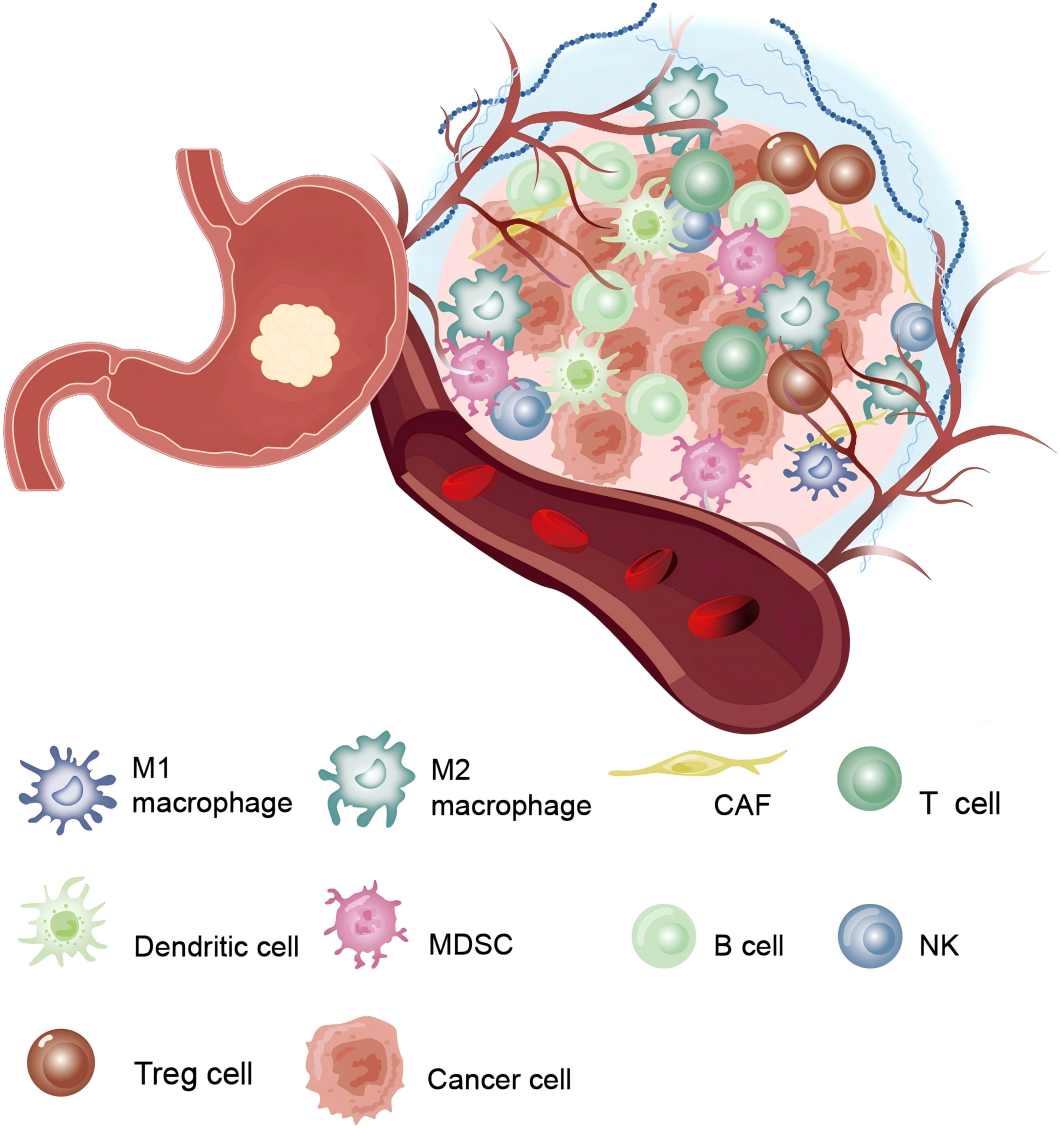
The tumor microenvironment in gastric cancer: Shows the complex interactions between various immune and non-immune cells surrounding the tumor.

The TME in GC represents a multifaceted and ever-evolving ecosystem (**Figure 2**). It comprises a diverse array of cellular components such as cancer-associated fibroblasts (CAFs), immune cells, and endothelial cells, alongside non-cellular factors like the extracellular matrix (ECM) and cytokines. This microenvironment is vital for the growth and progress of GC (16–18).

### 2.1 Cellular components

CAFs are the major stromal cells in the TME and promote Epithelial-Mesenchymal Transition (EMT) and tumor cell invasion and metastasis by secreting soluble factors (e.g., chemokines, cytokines, growth factors TGF-β, and VEGF) and remodeling the ECM (19, 20).

Immune cells demonstrate a significant bidirectional regulatory role, crucial in modulating tumor progression, immune evasion, and resistance to therapy (21). TAMs (Tumor−associated macrophages) are a crucial immune cell component within the GC TME. They exhibit distinct phenotypes, with the M1 phenotype displaying anti-tumor properties and the M2 phenotype promoting tumor progression (22). The M2-type TAMs enhance tumor progression by secreting pro-angiogenic factors, such as VEGF, and immunosuppressive cytokines, including IL-10 and TGF-β. Additionally, the buildup of regulatory T cells (Tregs) and myeloid-derived suppressor cells (MDSCs) notably hinders the immune response of effector T cells against tumors, thereby worsening the issue of immune evasion in GC (20). T cells are essential in restricting tumor growth during the immune editing process (23), and can be classified into CD4+ T cells and CD8+ T cells. The anti-tumor effects of CD8+ T cells are often hindered by various immunosuppressive pathways, such as the overexpression of immunosuppressive factors by cancer cells and components within the TME. The activation of these immune checkpoints limits T cell proliferation and cytotoxic activity, significantly reducing the efficacy of immunotherapy (14, 20). The role of T cells has been thoroughly investigated and implemented in clinical practice. While some immunotherapy approaches targeting B lymphocytes have shown therapeutic benefits in certain studies, several challenges and limitations persist. In most GC, infiltrating tumor-associated B cells are found in the form of tertiary lymphoid structures (TLS), located both around the tumor and in the surrounding normal gastric mucosa (24). TIGIT+ B cells can influence immune-infiltrative structures to drive tumor progression, whereas MALT-B cells activate the complement pathway, thereby boosting anti-tumor immunity (25, 26). However, research on B cells in cancer is limited, and further investigation into the mechanisms of B cells in cancer, along with the exploration of new immunotherapy strategies, is of significant importance to improve cancer treatment outcomes. Natural killer (NK) cells can identify target and eliminate cells, recognizing tumor cells that may evade detection by CD8+ T cells. The level of NK cell infiltration in both tumors and peripheral blood is strongly linked to the prognosis of GC patients (27, 28). GC cells produce prostaglandin E2 (PGE2), which inhibits the proliferation of NK cells and promotes their apoptosis (27). Research has demonstrated that enhancing the cytotoxic capacity of NK cells can inhibit immune evasion in GC (29). Currently, research on NK cells is relatively limited, and there is hope for more controlled trials in the future to validate their role. Dendritic cells (DCs) are the most powerful antigen-presenting cells, proficient in efficient antigen cross - presentation. They are essential for antitumor immunity by imodulating the TME and recruiting and activating anticancer T cells (30). Therefore, by hindering DC activation, antigen presentation, maturation, recruitment, and differentiation, both the TME and GC cells can evade immune surveillance (31). The plasticity of immune cells and their interactions with tumor cells establish the immunosuppressive characteristics of the TME and play a vital role in therapeutic resistance. Moreover, different subtypes of the TME exhibit significant heterogeneity. MSI-H and EBV-positive gastric esophageal adenocarcinomas (GEA) are typically associated with notable T cell infiltration, whereas the GS subtype is enriched with CD4+ T cells, macrophages, and B cells. In the GS subtype, TLS are present in about 50% of cases. In contrast, CIN-type cancers are characterized by CD8+ T cell infiltration at the tumor periphery, alongside active infiltration by tumor-associated macrophages (32, 33). Currently, there are relatively few studies on B cells and NK cells, and the immune escape mechanism is still not fully understood. Future development could focus on breakthroughs in immune escape mechanisms and in-depth studies of immune cell interactions in the TME.

Endothelial cells are a key element of the TME in GC, playing a crucial role in tumor angiogenesis as well as the regulation of tumor progression and metastasis (34). In GC, increased angiogenesis is generally linked to a worse prognosis. This process is driven by a variety of pro-angiogenic factors, including VEGF, fibroblast growth factor (FGF), and platelet-derived growth factor (PDGF) (35). Compared to normal endothelial cells, endothelial cells within the TME exhibit significantly distinct phenotypic and functional characteristics (36). Tumor-associated endothelial cells (TEC) typically possess enhanced proliferative capacity, migratory ability, and permeability. They also express unique surface markers and adhesion molecules, which collectively facilitate the extravasation of cancer cells and their subsequent distant metastasis (37).

### 2.2 Non-cellular components

The extracellular matrix (ECM) is a dynamic and intricately organized tissue structure. The onset and progression of GC are accompanied by significant remodeling of the ECM, including the overexpression of collagen, fibronectin, and laminin, which collectively enhance the tumor’s invasive potential (38, 39). Matrix metalloproteinases (MMPs) in the ECM are crucial for degrading the basement membrane and stroma, thereby facilitating the spread and encroachment of cancer cells (39). The ECM’s role in GC is reflected in its remodeling of the TME and its regulation of cancer cell behavior, making it a promising target for therapeutic intervention. Future research focusing on the ECM in GC could lead to innovative and more effective treatment strategies. The expression levels of certain cytokines are linked to the onset, invasion, metastasis, and prognosis of GC (40). Studies have suggested that tumor necrosis factor-alpha (TNF-α), IL-37, GDF15, CXCR2, and other factors may serve as potential therapeutic agents (41–44). Ongoing investigation of cytokines shows potential for creating innovative, targeted treatments for GC (45).

Most studies have focused on the role of specific cytokines or molecules, with less research on how these factors synergize in the complex TME, which needs to be further explored for their combined therapeutic effects and clinical validation. Future studies should focus on targeting ECM and cytokines.

## 3 Perioperative immunotherapy

### 3.1 Immunotherapy plus chemotherapy

For patients with locally advanced gastric cancer (AGC), surgical intervention remains the key approach for achieving disease cure. However, because of the significant risk of postoperative local recurrence and metastasis, surgery alone is insufficient to achieve satisfactory outcomes. Perioperative chemotherapy or chemoradiotherapy is considered the standard treatment for early-stage GC. Neoadjuvant chemoradiotherapy based on the CROSS regimen has demonstrated a pathological complete response (pCR) rate of nearly 30% (46). In gastric or gastroesophageal junction (G/GEJ) adenocarcinomas, preoperative and postoperative FLOT chemotherapy regimens also showed improved overall survival (OS) outcomes compared to perioperative ECF/ECX (47). There is still significant room for improvement in pCR and OS in the perioperative setting. While the pCR rate for perioperative treatment remains relatively low, further advancements in OS face numerous challenges. Enhancing patient survival and response rates, either preoperatively or postoperatively, has become a focal point of current research. In recent years, ICIs, as an emerging therapeutic approach, have demonstrated considerable potential in addressing multiple cancer types. The application of ICIs in the perioperative treatment of GC may offer patients additional treatment options and significantly improve clinical outcomes (**Table 1**).

**Table 1:** Overview of Clinical Trials on Perioperative Immunotherapy for Gastric Cancer.

Clinical trial	n	Target	Phase	Drug	Patient selection	primary end point	Treatment-related adverse event rates	
							Severe or life-threatening adverse events grade ≥3	Leading to death
CheckMate 577 (48)	532	PD-1	III	Nivolumab vs Placebo	post-operative	Median DFS:22.4vs11.0	71 (13%) vs 15 (6%)	The trial regimen was discontinued.
KEYNOTE-585 (49)	1254	PD-1	III	Pabolizumab plus cisplatin vs placebo plus cisplatin	Perioperative	Median EFS:44.4vs25.3	312 (78%)vs 297 (74%)	4 (1%)vs 2 (<1%)
MATTERHORN (50)	900	PD-L1	III	Durvalumab	Perioperative	EFS: Ongoing	–	–
ICONIC (52)	44	PD-L1	II	Avelumab plus FLOT	Perioperative	pCR not reached 25%	–	–
GASPAR (53)	67	PD-1	II	Spartalizumab plus FLOT	Perioperative	Ongoing	–	–
NCT05715632 (54)	46	PD-1	II	Camrelizumab	Perioperative	one-year EFS rate:93.1%	9 (19.6%)	0
NEOSUMMIT-01 (55)	108	PD-1	II	Toripalimab plus SOX/XELOX	Perioperative	Close to the complete remission rate:44.4%	35.2%	1.9%
NEONIPIGA (57)	32	PD-1+CTLA-4	II	Nivolumab plus ipilimumab	Perioperative (dMMR/MSI-H)	pCR (pathological T0N0):58.6%	6 (19%)	1
INFINITY (58)	310	PD-L1+CTLA-4	II	Tremelimumab and durvalumab	Perioperative (MSI)	pCR:60%	–	2
PANDA (60)	21	PD-L1	II	Atezolizumab	Perioperative(HER2+)	Median DFS was not reached	2 (10%)	–
NCT03950271 (61)	22	PD-1	II	Carelizumab + Trastuzumab+CAPOX	Perioperative(HER2+)	pCR:31.3%, R0 Resection Rate: 100%	–	0
*Chai* et al. (62)	23	PD-1	II	Vidisicumab + Carelizumab+ S-1	Perioperative(HER2+)	MPR:50%, R0 Resection Rate: 100%	–	0

The CheckMate 577 trial evaluated the efficacy of ICIs in patients with stage II and III esophageal or GEJ cancer who, after undergoing neoadjuvant chemoradiotherapy and surgery, still presented with residual pathological disease (48). After one-year treatment and a median 24.4 - month follow - up, the nivolumab group had a median disease-free survival (DFS) of 22.4 months, compared to 11 months in the placebo group. Nivolumab was significantly associated with improved DFS, lowering the risk of recurrence or death by 31%. In the primary cohort of the phase III KEYNOTE-585 trial, the pCR rates were significantly higher in the chemoimmunotherapy arm compared to the chemotherapy-alone arm (12.9% vs. 2.0%; P<0.001). Within the FLOT subpopulation, patients receiving pembrolizumab plus FLOT chemotherapy achieved a superior pCR rate versus those receiving placebo plus FLOT (17% vs. 7%) (49). The observed heterogeneity in outcomes across cohorts may be attributable to differences in chemotherapy regimens. Notably, only 20% of participants received the standard perioperative FLOT regimen in this trial, with the majority having been exposed to suboptimal chemotherapy protocols prior to enrollment. These findings warrant further validation in subsequent clinical trials. In response, the MATTERHORN trial explored the combination of immunotherapy with perioperative FLOT, comparing durvalumab plus FLOT chemotherapy followed by adjuvant durvalumab monotherapy in patients with resectable G/GEJ cancer(G/GEJC) (50). In an interim analysis, the pCR rate was 19% in the durvalumab group and 7% in the placebo group, with an absolute inter-arm difference of 12%, showing some improvement. However, the results for event-free survival (EFS) are still pending, and there is inadequate evidence to justify the regular use of ICIs in the perioperative context.

In comparison to concurrent immunotherapy or postoperative PD-L1 inhibition, the administration of anti-PD-L1 antibodies prior to chemotherapy significantly increases the infiltration of CD8+ T cells and demonstrates a stronger antitumor response (51). Although the ICONIC trial did not achieve a pCR rate of 25%, it reported a promising 12-month progression-free survival (PFS) of 93.1% (52). Trials such as GASPAR have also focused on perioperative immunotherapy combined with chemotherapy for GC (53). Additionally, there has been progress in combining other chemotherapy regimens with ICIs. In a phase II trial (NCT05715632), camrelizumab was used in combination with CAPOX for four cycles, followed by surgery and an additional four cycles of camrelizumab combined with CAPOX (54). Nine patients achieved pCR, 25 patients had major pathological responses, and the objective response rate was 69.6%, with both the 1-year EFS and DFS rates at 93.1%. This trial demonstrated that the perioperative camrelizumab plus CAPOX regimen shows good pathological response in resectable locally advanced G/GEJ adenocarcinoma patients, though it is limited by a small sample size. Other phase II trials, such as NEOSUMMIT-01, are also exploring the use of different ICIs and chemotherapy regimens in the perioperative setting, with hopes for further advancements in this area (55).

In the KEYNOTE-585 trial, subgroup analyses of EFS and OS revealed that patients with combined positive score (CPS) > 10, Microsatellite instability-High (MSI-H), and PD-L1 expressing tumors benefited more from the treatment (49). MSI status serves as a crucial prognostic indicator and a predictive biomarker for therapeutic response in GC (56). Trials such as NEONIPIGA (57) and INFINITY (58), which have explored the use of ICIs in the perioperative setting for MSI-H populations, have shown promising results. However, these trials had limited sample sizes, and larger studies are required to confirm these findings. Currently, the research on perioperative immunotherapy is gradually increasing; however, existing studies have yet to provide sufficient evidence to alter clinical practice. Future large-scale randomized controlled trials are essential to validate its clinical efficacy and generate more robust evidence for its use in clinical practice.

### 3.2 Immunotherapy plus anti-HER2 therapy

Building upon perioperative immunotherapy, the strategy of combining anti-HER2 treatment with immunotherapy offers a new therapeutic direction for HER2-positive GC patients. Trastuzumab is a drug that targets HER2, and trastuzumab in combination with chemotherapy is the standard first-line treatment option for HER2-positive advanced G/GEJC. The ToGA trial was the first to establish the substantial survival advantage of trastuzumab combined with chemotherapy for advanced HER2-positive GC, thereby setting the groundwork for the use of perioperative anti-HER2 therapies (59). In the second phase of the PANDA trial, 21 treatment-naive patients with resectable G/GEJC initially underwent one cycle of atezolizumab monotherapy, followed by four cycles of atezolizumab combined with docetaxel, oxaliplatin, and capecitabine (60). Among the 20 patients who underwent surgery, 14 achieved remission, and 13 maintained disease-free survival; among the 6 non-responders, 5 died due to cancer-related causes. Additionally, compared to trastuzumab combined with CAPOX alone, the pCR rate was higher in HER2 - positive, locally advanced, resectable G/GEJC patients who received atezolizumab (38.1% vs. 14.3%, P=0.079). Another phase II study yielded similar results, wherein 22 HER2-positive G/GEJC patients were treated with neoadjuvant treatment combining camrelizumab, trastuzumab, and CapOX (61). The results showed a pCR rate of 31.3% and an R0 resection rate of 100%. A prospective, single-center, single-arm phase II clinical trial included 23 patients with HER2-overexpressing locally advanced resectable G/GEJC to assess the efficacy and safety of RC48 in combination with camrelizumab and S-1 as a neoadjuvant treatment. The results showed a 100% R0 resection rate in patients who underwent surgery, with 6 patients (50%) achieving major pathological response (MPR), including 4 (33.3%) achieving pCR, with good safety (62). However, these trials are limited by small sample sizes, which may not be fully representative. Larger-scale randomized controlled trials are necessary to clarify the advantages of perioperative combined treatment strategies using ICIs and to determine whether HER2-positive patients receive greater benefits. In the INNOVATION trial, trastuzumab combined with patuximab could exert synergistic anti-tumor effects through dual blockade of the HER2 signaling pathway (63). In this regard, the combination strategy of dual-targeted therapy and immunotherapy can be further explored in the future.

## 4 Immune checkpoint inhibitor therapy

### 4.1 Mechanism

Tumor cells evade immune detection and continue to grow by aberrantly expressing immune checkpoint molecules that inhibit T cell activation, thereby achieving immune escape. ICIs boost the body’s anti-tumor immune response by inhibiting signals that suppress T cell activation (64). As a form of cancer immunotherapy, ICIs target specific immune receptors located on the surface of T lymphocytes to enhance anti-tumor immune responses (65). T cell activation relies on the presentation of antigens by major histocompatibility complex (MHC) class II molecules on antigen-presenting cells (APCs) along with co-stimulatory signals. Cytotoxic T-lymphocyte-associated protein 4 (CTLA-4) primarily functions as a negative regulator during the early stages of T cell activation (66). Anti-CTLA-4 antibodies promote further T cell activation by blocking the inhibitory signals from CTLA-4 binding (67). The interaction between the PD-1 receptor and its ligand PD-L1 induces T cell apoptosis, and anti-PD-1/PD-L1 antibodies inhibit this signaling pathway to prevent tumor immune escape (68). PD-1 and CTLA-4 serve as co-inhibitory receptors on T cells, facilitating immune evasion by tumor cells through the negative regulation of T cell function (69). ICIs target these receptors to reinvigorate the anti-tumor immune response. In studies of AGC, ICIs have demonstrated significant efficacy, driving the development of immunotherapy (70, 71) (**Table 2**). Furthermore, antibodies targeting other ICIs, such as LAG3, ICOS, TIGIT, TIM3, GITR, and 4-1BB, are actively being explored in clinical trials (72), with hopes for the development of additional ICIs for cancer treatment in the future.

**Table 2:** Overview of clinical trials on immune checkpoint inhibitors in gastric cancer.

Line	Clinical Trial	n	Target	Phase	Drug	Patient Selection	ORR(%)	Median OS (months)	Median PFS (months)	Treatment-related adverse event rates			
										grade ≥3	Common Adverse Reactions	Leading to discontinuation	Leading to death
First-line	KEYNOTE-062 (73)	763	Her2-	III	Pembrolizumab vs chemotherapy	PD-1	–	CPS ≥1: 10.6 vs 11.1 CPS ≥10: 17.0 vs 10.8	CPS ≥1: 2.0 vs 6.4 CPS ≥10: 2.9 vs 6.1	17% vs 69%	Nausea, fatigue, among others.	10 (3.9%) vs 44(18.0%)	3 vs 3
					Pembrolizumab Plus chemotherapy			CPS ≥1: 12.5 CPS ≥10: 12.3	CPS ≥1: 6.9	73%		69 (27.6%)	5
	JAVELIN Gastric 100 (71)	499	Her2-	III	Avelumab vs chemotherapy	PD-L1	–	10.4vs10.9	–	12.8% vs 32.8%	Elevated amylase, lipase, among others.	25 (10.3%) vs 65 (27.3%)	0 vs 1
	NCT02954536 (82)	37	HER2+	II	Pembrolizumab + trastuzumab + chemotherapy	PD-1	91	27.2	13	67%	Paresthesia or peripheral sensory neuropathy, among others.	4	0
	KEYNOTE-811 (83)	692	HER2+	III	Pembrolizumab + trastuzumab + CF/CAPOX/SOX	PD-1 + HER2	74.4	–	–	57.1%	–	24.4%	3.2%
	CP-MGAH22-05 (86)	95	HER2+	Ib/2	Pembrolizumab + margetuximab	PD-1	18.48	–	–	9%	Anaemia,infusion-related reactions, among others.	–	0
	MAHOGANY (87)	43	HER2+ and CPS ≥ 1	III	Retifanlimab/ tebotelimab + margetuximab	PD-1 (+LAG-3) + HER2	64.8	–	–	–	Infusion related reaction, diarrhea and fatigue, among others.	1	–
	INTEGA (88)	88	HER2+	II	Ipilimumab + nivoluamb	CTLA-4 + PD-1 + HER2	32	16.4	3.2	82%	Anemia, infection, and diarrhea.	21%	5
	PANTHERA (85)	43	HER2+	Ib/2	Pembrolizumab+trastuzumab + two-drug chemotherapy	PD-1+ HER2	76.7	19.3	8.6	83.7%	neutropenia, anemia, and diarrhea.	16	1
	KEYNOTE-659 (91)	54	Her2-	IIb	Pembrolizumab plus chemotherapy	PD-1	72.2	Not reached	9.4	57.4%	Decreased platelet count,reduced neutrophil count,among others.	40	0
	KEYNOTE-859 (92)	1579	Her2-	III	Pembrolizumab plus chemotherapy vs placebo plus chemotherapy	PD-1	–	ITT:12.9 vs 11.5 CPS ≥1:13.0vs 11.4 CPS≥10:15.7 vs 11·8	–	12% vs10%	Anemia, reduced neutrophil count,among others.	–	8 (1%) vs 16(2%)
	CheckMate 649 (97)	1581	Her2-	III	Nivolumab plus chemotherapy vs chemotherapy	PD-1	51vs41	14.4vs11.1	7.7vs6.1	22%vs 12%	Nausea, diarrhea, and peripheral neuropathy.	284 (36%)vs 181 (24%)	16 (2%) vs4(1%)
	ATTRACTION-4 (98)	40	Her2-	II	Nivolumab plus SOX vs Nivolumab plus CAPOX	PD-1	57.1vs76.5	Not reached	9.7vs10.6	57.1% vs66.7%	Neutropenia,anemia, among others.	3 (14.35)vs 2 (11.1%)	0
		724		III	Nivolumab plus chemotherapy vs placebo plus chemotherapy		–	17.45vs17.15	10.45vs8.34	25% vs 14%	Neutropenia, thrombocytopenia,among others.	–	3vs3
	CTR20181270 (93)	35	All	Ib	HX008 combined with oxaliplatin plus capecitabine	PD-1	60	NR	9.2	11.4%	Anemia, neutropenia, among others.	26 (74.3%)	1(2.9%)
	ORIENT-16 (94, 95)	650	Her2-	III	Sindilizumab plus chemotherapy	PD-1	58.2	15.2 CP≥ 5:18.4	7.1	59.8%	Decreased platelet count, among others.	48.3%	6(1.8%)
	RATIONALE-305 (96)	99	Her2-	III	Tislelizumab plus chemotherapy VS placebo plus chemotherapy	PD-1	–	17.2	–	54%vs50%	Decreased neutrophil count,thrombocytopenia,among others.	80 (16%)vs 40 (8%)	6 (1%)vs 2 (<1%)
	RELATIVITY-060 (106)	274	All	II	Nivolumab and Relatlimab Plus Chemotherapy	PD-1+LAG-3	48	13.5	7	69%	neutropenia, fatigue, among others.	42%	3
	GEMSTONE 303 (100)	479	CPS ≥ 5	III	Sugemalimab plus CAPOX	PD-L1	–	15.6	7.6	53.9	–	–	–
	AK104-201 (101, 102)	96	All	Ib/II	AK104 plus CAPOX	PD-1/CTLA-4	68.2	17.41	9.2	69.4	platelet count decreased, among others.	–	–
Second-line	KEYNOTE-061 (75)	592	CPS ≥ 1	III	Pembrolizumab vs Paclitaxel	PD-1	–	9.1vs8.3	1.5vs4.1	14%vs35%	Anemia, peripheral neuropathy,among others.	9(3%)vs 15(5%)	3(1%)vs1(<1%)
Second or later line	KEYNOTE-059 (77)	259	All	II	Pembrolizumab	PD-1	15.5	5.6	2	17.8%	Fatigue, pruritus, rashes, among others.	2	2
	NCT04280341 (89)	56	HER2+vs low HER2 expressing	I	Tislelizumab+RC48	PD-1+ADC	56vs46	NEvs14	7.8vs5.1	59%(all subjects)	Decreased white blood cell count, among others.	8	1
	*Yan* et al. (90)	57	HER2 2+/3+	retrospective study	PD-1+RC48	PD-1+ADC	41.7	13.2	5.8	41.7%	–	–	0
	CheckMate 032 (103)	160	All	II	Nivolumab plus Ipilimumab vs Nivolumab	PD-1 ± CTLA-4	24vs12	6.9vs6.2	1.6vs1.4	43%vs10%	Fatigue, pruritus, among others.	20%vs3%	0
Third-line	JAVELIN Gastric 300 (78)	371	All	III	Avelumab vs chemotherapy	PD-L1	2.2vs4.3	4.6vs5.0	1.4vs2.7	9.2%vs31.6%	Nausea, diarrhea, among others.	7 (3.8%)vs 9 (5.1%)	0vs1
Third or later-line	ATTRACTION-2 (79)	493	All	III	Nivolumab vs placebo	PD-1	11.2vs0.0	5.26vs4.14	1.61vs1.45	10% vs 4%	Pruritus, diarrhea, among others.	9 (3%) vs 4 (2%)	5 (2%) vs 2(1%)

### 4.2 Anti-PD-1/PD-L1 antibody monotherapy

#### 4.2.1 First-line therapy

The phase III KEYNOTE-062 trial sought to assess the effectiveness and safety of pembrolizumab versus chemotherapy as a first-line therapy for AGC (73). Even though Pembrolizumab monotherapy didn’t show a marked OS edge over chemotherapy among patients with a CPS of at least 1 (median OS was 10.6 months compared to 11.1 months, HR = 0.91), it did have superior efficacy compared to chemotherapy in the subgroup of patients with a CPS of 10 or more. In this subgroup, Pembrolizumab had a median OS of 17.4 months, whereas chemotherapy had a median OS of 10.8 months (HR = 0.69), showing a 31% decrease in the death risk. These findings suggest that immunotherapy monotherapy may present an effective treatment option in patients with high CPS scores. The global open-label phase III trial JAVELIN Gastric 100 compared the efficacy of avelumab following induction chemotherapy with ongoing chemotherapy (74). In contrast to chemotherapy, avelumab monotherapy did not improve OS, but it demonstrated a more tolerable safety profile compared to chemotherapy. Furthermore, in an exploratory subgroup analysis, patients with CPS ≥1 and the 22C3 antibody were more likely to benefit from avelumab treatment. However, the trial lacked a comprehensive stratification based on PD-L1 CPS, potentially overlooking more precisely identified populations that could benefit from treatment.

The aforementioned experimental results highlight the current limitations of monotherapy with immuno-oncology agents. Future research should focus on the precise identification of populations that stand to benefit, the optimization of combination therapy strategies, the exploration of novel immunotherapeutic agents, and the conduct of large-scale clinical trials. These efforts are essential to further enhance the efficacy of immunotherapy and broaden its clinical applicability.

#### 4.2.2 Second or later-line therapy

The KEYNOTE-061 study aimed to evaluate the efficacy of pembrolizumab in comparison to paclitaxel for patients suffering from advanced G/GEJC, particularly those who had already been treated with chemotherapy based on platinum and fluoropyrimidines (75). The findings indicated a median OS of 9.1 months in the pembrolizumab group, whereas the paclitaxel group had a median OS of 8.3 months. Like the KEYNOTE-062 trial, the Kaplan-Meier OS curves for both groups intersected, suggesting heterogeneous prognostic outcomes. Moreover, even though in the second-line treatment of advanced G/GEJC patients with a PD-L1 CPS of at least 1, pembrolizumab didn’t bring about a significant improvement in OS when compared to paclitaxel, it had a much better safety record. Fuchs and his colleagues carried out the KEYNOTE - 059 trial, which showed that pembrolizumab had some clinical effectiveness in patients with AGC whose disease has progressed after 2 or more lines of therapy. The objective response rate (ORR) was 11.6% (76). In patients with PD-L1 positivity and a CPS ≥1, the ORR reached 15.5%. In the KEYNOTE-059 trial, *Bang* et al. (77) assessed the results from cohorts 2 and 3 of first-line treatment and confirmed that pembrolizumab, both as monotherapy and in combination with chemotherapy, showed good tolerability. In the randomized phase III JAVELIN Gastric 300 study (78), involving 371 patients with advanced G/GEJC, the safety and efficacy of avelumab were compared to chemotherapy. The median OS for the avelumab and chemotherapy groups was 4.6 months and 5.0 months, respectively, while the median PFS for the two groups was 1.4 months and 2.7 months. In the third-line setting, monotherapy with avelumab did not result in improved OS or PFS compared to chemotherapy. Nevertheless, avelumab exhibited a more tolerable safety profile compared to chemotherapy. In the randomized phase III ATTRACT-02 trial (79), the effects of nivolumab versus placebo were compared in patients diagnosed with unresectable or recurrent gastric adenocarcinoma or GEJC. The findings showed that the nivolumab group’s median OS was 5.26 months compared to 4.14 months for the placebo group, resulting in a HR of 0.63 (95% CI, 0.51–0.78; P<0.0001). *Chen* et al. (80) provided a 2-year data update from the ATTRACT-02 study, which showed that nivolumab significantly improved the one- and two-year OS rates compared to placebo, with rates of 27.3% versus 11.6% and 10.6% versus 3.2%, respectively. Nivolumab demonstrated more than a threefold increase in the two-year OS rate for patients undergoing third-line treatment for AGC. The ATTRACT-02 study established a solid foundation for the use of nivolumab in third-line and later-line treatment of AGC.

ICIs have demonstrated certain efficacy and a favorable safety profile in the second-line and later-line treatment of AGC, particularly in patients with PD-L1 CPS ≥1. However, the improvement in OS with monotherapy is limited, and the future should be devoted to optimizing combination therapy, focusing on special populations as well as developing individualized treatments.

### 4.3 Anti-PD-1/PD-L1 antibodies plus anti-HER2 therapy

#### 4.3.1 First-line therapy

Both preclinical and clinical studies have demonstrated that combining ICIs with anti-HER2 therapies can generate a synergistic effect in the treatment of HER2-positive GC (81). The NCT02954536 trial assessed the safety and effectiveness of the combination of pembrolizumab, trastuzumab, and chemotherapy as a first-line therapy for HER2-positive metastatic gastroesophageal cancer (GEC) (82). The results showed that pembrolizumab could be safely combined with trastuzumab and chemotherapy, demonstrating significant activity in HER2-positive metastatic GEC. Subsequently, the mid-term analysis of the large phase III KEYNOTE-811 study was presented at the 2021 ASCO conference. This study focused on treatment-naive HER2-positive AGC patients, demonstrating that the incorporation of pembrolizumab into the standard first-line treatment regimen resulted in a 22.7% improvement in the ORR (83). In patients with PD-L1 CPS ≥1, adding pembrolizumab to trastuzumab combination chemotherapy resulted in improved PFS compared to trastuzumab combination chemotherapy. The final OS analysis revealed a notable enhancement in OS for the pembrolizumab group in contrast to the placebo group, with a median OS of 20.0 months versus 16.8 months. Among participants having PD - L1 CPS ≥1, the pembrolizumab group had a median OS of 20.1 months, compared to 15.7 months in the placebo group, showing a statistically significant OS improvement (84). The single-arm, multicenter phase Ib/II PANTHERA study showed that first-line treatment with pembrolizumab combined with trastuzumab and chemotherapy in HER2-positive AGC yielded a median OS of 19.3 months and a median PFS of 8.6 months, with an ORR of 76.7% (85). The phase I/II CP-MGAH22–05 trial assessed the safety of margetuximab in conjunction with pembrolizumab in HER2-positive patients, reporting an ORR of 18.48%, which indicates a clinical benefit (86). The MAHOGANY trial explored the safety of margetuximab in combination with retifanlimab and tebotelimab. In the margetuximab + retifanlimab cohort, the ORR was 64.8%, demonstrating promising anti-tumor efficacy (87). The INTEGA trial delved into the effects of pairing trastuzumab and nivolumab with either FOLFOX or ipilimumab in HER2 - positive GEC. In the ipilimumab cohort, the 12-month OS rate stood at 57%, with a median OS of 16.4 months. Meanwhile, the FOLFOX group boasted a 12-month OS rate of 70%. However, this trial had a small sample size and did not conduct an in-depth analysis of PD-L1 CPS or other biomarkers, which could impact the reliability of the results (88).

The combination of immunotherapy and anti-HER2 treatment has shown significant synergistic effects in HER2-positive GC, with several clinical trials providing preliminary confirmation of its anti-tumor activity and safety. However, existing studies have limitations, such as small sample sizes, inadequate biomarker analysis, and a shortage of long-term observational data. Future trials should be conducted to further enhance the efficacy of combining immunotherapy with anti-HER2 therapies, ultimately providing patients with better treatment options.

#### 4.3.2 Second or later-line therapy

In 2024, findings of a phase I, multicenter, open-label, dose-escalating clinical trial were disclosed (89). This pioneering study explored the clinical efficacy of combining the HER2-targeting antibody-drug conjugates (ADC) RC48 with the PD-1 inhibitor toripalimab in patients with advanced HER2-positive and HER2 low-expressing GC. The study included 56 participants from three different research centers in China, with the primary objective of assessing the efficacy of RC48 combined with toripalimab as second-line or later treatment. The results revealed that in the HER2-positive G/GEJ adenocarcinoma patient group, the median PFS reached 7.8 months, with an ORR of 56%. However, the median OS data has not yet fully matured. These findings further support the potential synergistic effect between HER2-targeted ADCs and PD-1 inhibitors. In the same year, an observational, multicenter real-world study was presented at the ESMO GI Conference (90). The study enrolled patients with advanced G/GEJ adenocarcinoma who had previously received treatment for HER2 overexpression (IHC 3+ or 2+) and were treated with a combination of vidisitmab and a PD-1 inhibitor. Among these patients, 83% had previously received first-line immunotherapy. The study revealed that when vidisitmab was used in conjunction with a PD-1 inhibitor, the median OS hit 13.2 months, the median PFS stood at 5.8 months, and the ORR was 41.7%. This backed up the safety and effectiveness of pairing RC48 with PD-1 inhibitors. These studies offer new evidence supporting the combined use of ADCs and immunotherapy, demonstrating their potential in real-world clinical applications. Future research could involve collecting more real-world data to validate the applicability of clinical trial results to broader populations. Additionally, given the limited number of existing clinical trials combining immunotherapy and anti-HER2 treatments, further trials in this area are warranted to enhance our understanding of their clinical impact.

### 4.4 Anti-PD-1/PD-L1 antibodies plus chemotherapy therapy

In the phase IIb study KEYNOTE-659, *Kawazoe* et al. (91) evaluated 54 patients and found an ORR of 72.2%, demonstrating that the combination of SOX chemotherapy and pembrolizumab showed promising efficacy and manageable safety as a first-line treatment for advanced G/GEJC. In the KEYNOTE-859 study, pembrolizumab combined with chemotherapy significantly enhanced OS in HER2-negative AGC patients compared to the placebo group (92). In the KEYNOTE-062 study, the combination of pembrolizumab and chemotherapy achieved an OS of 12.5 months for patients with CPS ≥1, whereas pembrolizumab monotherapy yielded 10.6 months of OS. In patients with CPS ≥10, the OS was 12.3 months for the combination group versus 17 months for the monotherapy group. *Shitara* et al. (73) observed that patient benefits varied significantly based on PD-L1 expression. For patients with CPS ≥1, there was no clear benefit from ICIs-based combination chemotherapy, and for those with CPS ≥10, the combination therapy was even less effective than monotherapy. Although the study did not provide groundbreaking objective data, further analysis suggested that specific patient populations—such as those with high CPS expression or MSI-H—may derive more benefit from GC immunotherapy. Additionally, different combination regimens and drug choices may influence treatment efficacy.

In a phase Ib trial (CTR20181270) (93), the combination of HX008 with capecitabine and oxaliplatin was explored for its efficacy and safety in treating advanced G/GEJC. The results demonstrated an ORR of 60.0% and a disease control rate (DCR) of 77.1%. The interim report from the ORIENT-16 trial confirmed that the combination of sintilimab and chemotherapy provided significant OS benefits in patients with CPS ≥5 and across the entire population (94, 95). A global, multicenter phase III clinical trial, RATIONALE-305 (96), reported updated data showing that the PD-1 inhibitor tislelizumab in combination with chemotherapy significantly improved ORR, OS, and PFS in first-line treatment of AGC. PD-1 inhibitors, such as HX008 and camrelizumab, have shown promising clinical trial results, but limitations remain, including small sample sizes and the absence of long-term follow-up, which may not fully reflect the long-term efficacy and safety of these combination treatments. Future research should include larger-scale phase III trials, particularly multicenter, multinational studies, to further evaluate the efficacy and safety of HX008, camrelizumab, and other similar drugs in AGC and GEJC. Special focus should be given to evaluating differential efficacy across various patient subgroups.

CheckMate 649 (97) is a global, multicenter, randomized phase III trial that assessed the efficacy of nivolumab combined with chemotherapy as a first-line treatment for HER2-negative, advanced or metastatic GC, GEJC, and esophageal cancer (EC). In the GC patient population with a CPS ≥5, the nivolumab-chemotherapy combination demonstrated a substantial survival advantage compared to chemotherapy alone. This combination treatment extended OS from 11.1 months to 14.4 months (HR=0.68), while PFS increased from 6.0 months to 7.7 months, reducing the risk of disease progression or death by 32%. Notably, this survival benefit was observed across a broad range of patients, with similar improvements in OS (13.8 months vs. 11.6 months, HR=0.79) and PFS (7.7 months vs. 6.9 months, HR=0.79) in the overall population. Particularly in the Chinese subgroup analysis, patients receiving combination therapy in different CPS strata (≥5, ≥1, and the entire randomized population) showed clinically meaningful survival improvements: the median OS reached 14.3 months (compared to 10.3 months in the control group), and the median PFS was extended to 8.3 months (vs. 5.6 months in the control group). The survival benefit was most pronounced in patients with CPS ≥5, where the median OS in the combination group was nearly 6 months longer than in the control group (15.5 months vs. 9.6 months).

In the phase II ATTRACTION-4 trial, *Boku* et al. (98) confirmed that nivolumab combined with SOX/CAPOX showed good tolerability and promising efficacy in patients with unresectable, advanced, or recurrent HER2-negative G/GEJC. However, the latest data from the phase III ATTRACTION-4 trial showed that, compared to chemotherapy alone, the OS difference between the nivolumab-chemotherapy combination group and the chemotherapy-only group was not statistically significant, although the combination therapy showed a significant improvement in PFS (99). Nivolumab has become the first ICI approved for first-line treatment of GC in China. Despite its breakthrough efficacy in AGC treatment, nivolumab faces notable limitations, including high costs and a relatively high incidence of immune-related adverse events. It is hoped that future clinical trials will address these issues to improve the overall benefit-risk profile.

GEMSTONE 303 (100) is a randomized, double-blind phase III clinical trial aimed at evaluating the safety and efficacy of sugemalimab combined with CAPOX chemotherapy, compared to placebo plus chemotherapy. The results demonstrated that the combination of sugemalimab and chemotherapy improved median OS to 15.6 months, compared to 12.6 months in the placebo group. Median PFS was 7.6 months versus 6.1 months, with a 25% reduction in the risk of death for the sugemalimab group. In subgroup analyses, the sugemalimab-chemotherapy combination consistently showed significant clinical benefit compared to the placebo-chemotherapy group. However, there was no in-depth analysis of biomarkers such as PD-L1 CPS, MSI, or tumor mutational burden (TMB). Future studies focusing on PD-L1 inhibitors will be needed to further validate these findings and explore their potential in guiding treatment strategies based on molecular markers.

Cadonilimab is an innovative bispecific antibody immunotherapy that simultaneously targets both PD-1 and CTLA-4. In phase Ib/II clinical trials, researchers found that the combination of cadonilimab with CAPOX chemotherapy as a first-line treatment for AGC offered significant clinical benefits, especially for patients with low PD-L1 expression. In the AK104–201 study (101, 102), cadonilimab combined with chemotherapy was applied as first-line treatment for GC or GEJ adenocarcinoma, demonstrating notable long-term survival benefits.The study included patients with various CPS. In the overall patient population, the median OS reached 17.41 months, while the median PFS was 9.2 months, with a 12-month OS rate of 61.4% and an ORR of 68.2%. Notably, in patients with a CPS <5, significant efficacy was observed, with a median OS of 17.28 months and a median PFS of 7.23 months. For patients with CPS <1, the median OS was 17.64 months, and the median PFS was 8.18 months. In patients with CPS ≥5, the median OS was as high as 20.24 months, though the median PFS had not yet been reached. These preliminary findings indicate that bispecific antibodies directed against both PD-1 and CTLA-4 may offer a more promising therapeutic approach for patients exhibiting low PD-L1 expression. This discovery provides important insights for future therapeutic strategies, and future efforts should focus on further research into bispecific antibodies and the conduct of related clinical trials to validate and expand on these results.

### 4.5 Combination therapy with dual ICIs

The Checkmate-032 study compared nivolumab monotherapy with two ICIs combination therapy, nivolumab plus ipilimumab, in patients with advanced G/GEJC (103). The findings demonstrated that the combination therapy yielded a superior ORR and PFS when compared to nivolumab monotherapy. Another study found that low-dose ipilimumab combined with nivolumab improved the RR in PD-L1-positive GC patients, with mild adverse reactions (104). Tremelimumab, a human IgG2 monoclonal antibody that inhibits CTLA-4, has been evaluated in combination with durvalumab or as monotherapy for chemotherapy-refractory G/GEJC (105). While tremelimumab did not exhibit robust efficacy in all patients, some individuals experienced sustained anti-tumor effects, with OS exceeding 32.7 months. Relatlimab, an LAG-3 inhibitor, has been studied in various tumor types. The RELATIVITY-060 trial evaluated the combination of nivolumab and relatlimab with chemotherapy as a first-line treatment in treatment-naive patients with advanced G/GEJC (106). However, the study failed to achieve its primary endpoint. Currently, while the monotherapy efficacy of some inhibitors is limited, their combined use shows relative significance. Future strategies should focus on optimizing combination therapy regimens and, with the proliferation of assays such as single-cell and spatial grouping, better localize the spatial relationship between immune cells and tumors, later enabling the development of biomarkers based on individual patient characteristics and TME. This approach could lead to more precise and personalized immunotherapy treatment plans.

## 5 Other immunotherapies

### 5.1 Adoptive cell therapy

ACT involves the ex vivo activation of immune cells, followed by their expansion or modification in structure and function, before reintroducing them into the patient’s body to boost the immune response against tumors, aiding in the removal of tumor cells.

#### 5.1.1 Chimeric antigen receptor T-cell therapy

Chimeric Antigen Receptor T (CAR-T) cell therapy is highly specific and can be tailored for personalized treatment, and it has been utilized in clinical trials and therapeutic settings for treating various malignant tumors. In GC, clinical trials have explored CAR-T therapies targeting antigens such as HER2, carcinoembryonic antigen (CEA), and Claudin18.2. Engineered CAR-T cells targeting HER2-positive tumor cells have demonstrated significant antitumor efficacy in GC mouse models (107). Preclinical experiments have demonstrated that expanded CAR-T cells recognize HER2 antigens through an MHC-independent mechanism, activating and promoting the proliferation of central memory T cells. This process effectively leads to the eradication of HER2-positive GC cells derived from patients (108). CEA is a common tumor marker in gastrointestinal cancers, typically exhibiting aberrant expression in GC. CAR-T cell therapy targeting CEA is currently in the clinical trial phase. The primary aim of the clinical trial (NCT02349724) is to evaluate the safety of CEA-targeted CAR-T cells and determine the optimal infusion dose; however, no results have been reported to date. Claudin18.2 (CLDN18.2) is currently the most extensively studied target in CAR-T research for GC. CLDN18.2, a tight junction protein, is selectively expressed in cancer cells and minimally in normal tissues, making it an attractive candidate for targeted therapy (109). *Wang* et al. found a significant correlation between CLDN18.2 expression and OS. Low expression of CLDN18.2 in tumor tissues has been recognized as an independent prognostic indicator for GC patients (110). Novel bispecific Trop2/PD-L1 CAR-T cells are capable of targeting both Trop2 and PD-L1, as well as blocking immune checkpoints, thereby improving the antitumor effectiveness of CAR-T cells against GC (111). One study has shown that M28z10 T cells exhibit potent antitumor activity, representing a promising therapeutic strategy for GC (112). In recent years, researchers have developed and designed various specific CAR-T cells for the management of GC. Although the therapeutic efficacy for solid tumors has been less than optimal, these advancements have nonetheless provided new perspectives for immunotherapy.

#### 5.1.2 Other adoptive cell therapies

ACT therapy also encompasses TCR-T therapy, NK cell therapy, TIL(Tumor-Infiltrating Lymphocyte) therapy, CIK(Cytokine-Induced Killer) cell therapy, and DC therapy. TCR-T immunotherapy involves the introduction of TCR genes into peripheral blood T cells, enabling them to specifically recognize tumor antigens. TCR-T cells can proliferate more effectively in high antigen pressure environments and are not limited by the antigens present on the surface of target cells, allowing them to flexibly recognize a variety of different targets (113). Currently, TCR-T therapy has not achieved significant efficacy in GC; however, its ability to overcome the limitations imposed by the expression of target cell membrane antigens offers promising prospects in the field of immune cell therapy. TCRs with low binding affinity may exhibit improved safety by reducing off-target effects, but this could come at the cost of diminished tumor-killing potency. Conversely, high-affinity TCRs may enhance anti-tumor activity but carry a higher risk of unintended toxicity against healthy tissues (114). Several clinical trials are currently underway, and future efforts should focus on identifying additional antigens to provide new breakthroughs in the treatment of refractory tumors such as GC. NK cells play a pivotal role in innate immunity, demonstrating potent antitumor, antiviral, and antibacterial activity. They are capable of activating and modulating adaptive immune responses, contributing to the overall immune defense (115). Studies targeting HER2-positive GC have demonstrated that infusion of expanded and activated autologous NK cells, in combination with trastuzumab, significantly enhances the cytotoxicity of NK cells against trastuzumab-targeted cells (116). *Cao* et al. (117) investigated the efficacy of mesothelin (MSLN)-targeted CAR-NK cells in the treatment of GC. The experimental findings demonstrated that MSLN-targeted CAR-NK cells showed significant antitumor activity *in vitro*. NK cell therapy is a promising immunotherapeutic approach, but a key challenge lies in obtaining sufficient quantities of NK cells for clinical treatment, which remains a major hurdle for the widespread application of NK cell therapy. TIL therapy, an individualized form of tumor immunotherapy, has been shown to elicit sustained complete responses in refractory patients (118). Research indicates that tumor regression following TIL infusion is mediated by T cells reactive to new tumor antigens. These T cells, after ex vivo expansion, are reinfused, enhancing the therapeutic efficacy (119). Patients with GC who have a high density of TILs are linked to reduced tumor invasion depth, the absence of lymph node metastasis, earlier TNM staging, and significant improvements in PFS. Research has identified that intratumoral CD3+ TILs and pathological T staging are independent prognostic factors of clinical significance (120). However, the extended time required to generate an adequate quantity of TIL cells may limit the clinical applicability of this therapy (121). CIK therapy involves the isolation of mononuclear cells from peripheral blood, followed by cytokine-induced activation and expansion. A dual-arm, single-center trial investigated the effects of autologous CIK cell therapy combined with SOX (CIK-SOX) in patients with locally advanced or metastatic GC, comparing the outcomes with those of SOX treatment alone (122). The median PFS for the CIK-SOX group was 6.9 months, compared to 4.9 months for the SOX group (HR 0.80, p = 0.45). The median OS for the two groups was 17.8 months and 9.75 months, respectively (HR 0.76, p = 0.34). Although this treatment increased the duration of both PFS and OS, the results were not statistically significant, and further trials are needed for validation. The application of DCs therapy in GC is still in the exploratory phase, with hopes to bring new advancements to the immunotherapy of GC cells.

### 5.2 Cancer vaccines

Cancer vaccine therapy is categorized into preventive cancer vaccines and therapeutic cancer vaccines. These vaccines induce tumor regression by stimulating the patient’s immune response to specific tumor antigens (123). Analyses have suggested the potential of tumor vaccines for use in GC (124). In one trial, a small molecule toll-like receptor-7 agonist (T7) was coupled to monoclonal GC 7 antigen mono-epitope (T7-MG1) or tri-epitope (T7-MG3) to synthesize a vaccine via solid-phase synthesis using the Fmoc strategy. The results showed a significant increase in antigenic antibodies after vaccination, confirming the possibility of a GC vaccine design (125). A GC vaccine synthesized by covalent linkage of a TLR7 agonist to the GC antigen MG7-Ag tetra epitope was used to create a model of tumor attack by treating BALB/c mice in prophylactic or therapeutic vaccination schedules and using 5-FU combination therapy. The results showed that T7-MB immunization combined with 5-FU chemotherapy reduced tumor size and prolonged long-term survival (126). Moreover, nanostructured lipid carriers incorporating chlorine e6 as an *in situ* dendritic cell vaccine (NLC/Ce6) have demonstrated efficacy in suppressing the growth of both primary and metastatic GC (127). The complex preparation processes and frequent vaccination requirements have hindered the translation of many cancer vaccine strategies from laboratory settings to clinical application. In GC, most of the current research on tumor-specific vaccines is preclinical, and the potential effectiveness of tumor vaccines remains to be investigated.

### 5.3 Oncolytic virus therapy

Oncolytic viruses (OVs) are viruses, either occurring in nature or artificially created, intended to target and eliminate tumor cells (128). Preclinical studies have demonstrated that OVs can effectively control tumor growth by enhancing anti-tumor immune response through direct tumor lysis and killing (129). G47Δ is a third-generation oncolytic herpes simplex virus type 1 that demonstrated effective cytopathic effects and replication in nine human GC cell lines *in vitro*. These findings suggest that teserpaturev is promising for the treatment of GC (130). Studies show that CF33-OVs deliver functional proteins and exhibit potent anti-tumor activity in GCPM models (131). CF17 is a new replication-competent chimeric poxvirus. Another preclinical study demonstrated that CF17 effectively infects, replicates and kills GC cells *in vitro* in a dose- and time-dependent manner. *In vivo*, intraperitoneal CF17 treatment showed potent anti-tumor activity in an aggressive GCPM model (132). *Ishikawa* et al. demonstrated that the combination of attenuated adenovirus (OBP-401) and paclitaxel (PTX) inhibited peritoneal metastasis of GC, suggesting that PTX intravenous virotherapy may be a promising therapeutic strategy for peritoneal metastasis of GC (133). Additional clinical evidence is required to back the present application of OVs in GC.

## 6 Biomarkers

Biomarkers play a crucial role in predicting patient responses to ICI therapies, evaluating therapeutic efficacy, and monitoring resistance (134). MSI, TIM-3, and TMB are among the biomarkers that have garnered significant attention in recent GC research. These biomarkers not only possess potential value in the diagnosis and prognosis of GC but also show considerable promise in predicting responses to immunotherapy and guiding clinical applications. MSI-H GC patients typically exhibit a higher mutational burden, which enhances the recognition and attack of their tumors by the immune system, resulting in better responses to ICIs (135). A high TMB indicates a greater number of mutations within the tumor, thereby increasing its immunogenicity and facilitating its detection and destruction by the immune system (136). As a result, TMB is regarded as a crucial biomarker for forecasting the effectiveness of immunotherapy. Additionally, PD-L1 expression, Epstein-Barr virus (EBV) infection, and circulating tumor DNA (ctDNA) are also biomarkers associated with the treatment outcomes of GC (136), with further clinical trials anticipated to validate their relevance. In the future, personalized treatment strategies may be developed through the detection of specific biomarker expression levels, thereby enhancing therapeutic efficacy while minimizing adverse reactions. NCT06349967 Trial Shows TMB, MSI, PD-L1 Expression, TIL Profiles, and Gut Microbiota That Will Help Personalize Treatment and Identify Patients Most Likely to Benefit from Immune Rechallenge (137, 138). Ongoing research should focus on exploring the precise mechanisms of action of these biomarkers and their differential expression across various populations to better inform clinical practice and improve patient survival rates.

## 7 Future research directions in immunotherapy for GC

Immunotherapy shows great potential in treating GC and has achieved impressive effectiveness in recent years. Nevertheless, the effectiveness of immunotherapy in GC continues to encounter specific challenges and limitations. For example, monotherapy often has limited efficacy in GC immunotherapy. As a result, combining immunotherapy with conventional chemotherapy and targeted treatments has become a significant focus of research. By combining ICIs with chemotherapy agents, molecular-targeted drugs, and other therapies, synergistic effects can be achieved, enhancing the overall therapeutic impact. The immune microenvironment of GC is relatively complex, with immune cell dysfunction often present. Thus, modulating the GC TME to improve the tumor’s immunosuppressive state is a critical research direction for enhancing the effectiveness of immunotherapy. Immunotherapy resistance remains one of the major challenges, and future research should prioritize investigating the molecular foundations of these resistance mechanisms, while also developing novel strategies to overcome immune resistance.

The fundamental concept of cytokine therapy lies in utilizing cytokines to activate the immune system, thereby targeting and destroying tumor cells. This approach has been proposed for quite some time. In melanoma, the combination of recombinant human IL-2 and ipilimumab has demonstrated superior efficacy compared to ipilimumab alone, without increasing adverse reactions (139). Currently, recombinant IL-2 formulations are undergoing clinical trials at various stages for GC. Similarly, IFN-γ and TNF formulations have also entered different experimental phases. Bacterial immunotherapy employs bacteria or their derivatives to activate or modulate the host immune system, enhancing the body’s ability to recognize and eradicate tumor cells. However, there have been relatively few trials, and its application in GC remains limited. It is anticipated that future large-scale trials and clinical research will provide more insight into its potential.

Furthermore, micro/nanocarrier technologies facilitate the delivery of specific tumor antigens, immune activators, or therapeutic agents to tumor sites, thereby activating antitumor immune responses and promoting tumor cell destruction. *Liu* et al. (140) discovered that selenium-containing nanocarriers could enhance the expression of NKG2D on the surface of NK cells and increase the expression of NKG2DL on tumor cell surfaces, thus improving the ability of NK cells to recognize and kill tumor cells, and enhancing their antitumor immune effects. Additionally, 3D printing technology utilizes biomaterials to print specific antigen structures ex vivo. *Zang* et al. (141) developed a 3D-printed scaffold containing immune modulators, which could strengthen the antitumor immune response. Although research into micro/nanocarrier technologies has demonstrated their potential in targeted cancer therapy, several technical barriers still impede their clinical translation. The stability of carriers, the controllability of drug release, and the distribution and clearance mechanisms within the body require further optimization. The application of 3D printing technology in antitumor immunotherapy remains in its exploratory phase. While its innovation in printing specific antigen structures ex vivo is promising, its practical benefits in clinical settings remain uncertain. Future efforts should focus on clinical research to assess its potential more thoroughly.

## 8 Conclusion

Immunotherapy has demonstrated significant potential in the treatment of GC, particularly in advanced stages, where ICIs have shown preliminary clinical progress. However, the complex TME of GC, along with tumor heterogeneity, drug side effects, and other factors, limits the widespread efficacy of immunotherapy. Despite these challenges, ongoing research into the GC IME and the continuous optimization of precision immunotherapy strategies offer promise for enhancing therapeutic efficacy through personalized treatment approaches in the future.

Current challenges primarily focus on patient selection, the development of immune biomarkers, the optimization of combination therapies, and the improvement of treatment tolerance. Specifically, immunotherapy strategies targeting subgroups with MSI-H and EBV positivity have shown promising prognoses, suggesting that these patients may represent a potential advantageous group for immunotherapy. Additionally, perioperative immunotherapy is emerging as a novel direction in GC treatment. Although some clinical trial results indicate that combining immunotherapy with chemotherapy improves patient survival, sufficient evidence is still lacking to recommend its routine use in clinical practice.

Future research needs to further explore the combined effects of immunotherapy with other treatment modalities, optimize the dosing of immunotherapies, and validate their long-term efficacy and safety through large-scale, multi-center clinical trials. Particularly in the development of different biomarkers and personalized immunotherapy regimens, there is potential for a crucial impact on improving survival rates and quality of life for GC patients, offering new therapeutic options and hope for these individuals.

## Glossary

GC: Gastric cancer
IME: immune microenvironment
TME: tumor microenvironment
TAMs: Tumor−associated macrophages
ICIs: immune checkpoint inhibitors
CAFs: cancer-associated fibroblasts
ECM: extracellular matrix
EMT: Epithelial-Mesenchymal Transition
MDSCs: myeloid-derived suppressor cells
TLS: tertiary lymphoid structures
NK: Natural killer
PGE2: prostaglandin E2
CTLA-4: Cytotoxic T-lymphocyte-associated protein 4
DCs: Dendritic cells
GEA: gastric esophageal adenocarcinomas
FGF: fibroblast growth factor
TEC: Tumor-associated endothelial cells
ECM: extracellular matrix
MMPs: Matrix metalloproteinases
AGC: advanced gastric cancer
pCR: pathological complete response
G/GEJ: gastric or gastroesophageal junction
OS: overall survival
DFS: disease-free survival
G/GEJC: G/GEJ cancer
EFS: event-free survival
PFS: progression-free survival
CPS: combined positive score
TIL: Tumor-Infiltrating Lymphocyte
CIK: Cytokine-Induced Killer
MSI-H: Microsatellite instability-High
MPR: major pathological response
MHC: major histocompatibility complex
APCs: antigen-presenting cells
ORR: objective response rate
GEC: gastroesophageal cancer
ADC: antibody-drug conjugates
DCR: disease control rate
EC: esophageal cancer
TMB: tumor mutational burden
ACT: Adoptive cell therapy
CAR-T: Chimeric Antigen Receptor T
CEA: carcinoembryonic antigen
CLDN18.2: Claudin18.2
MSLN: mesothelin
CIK-SOX: CIK cell therapy combined with SOX
OVs: Oncolytic viruses
PTX: paclitaxel
EBV: Epstein-Barr virus
ctDNA: circulating tumor DNA
